# Matriptase and MET are prominently expressed at the site of bone metastasis in renal cell carcinoma: immunohistochemical analysis

**DOI:** 10.1007/s13577-014-0101-3

**Published:** 2014-09-04

**Authors:** Shoichiro Mukai, Kenji Yorita, Yukari Kawagoe, Yuichi Katayama, Kozue Nakahara, Toyoharu Kamibeppu, Satoru Sugie, Hiromasa Tukino, Toshiyuki Kamoto, Hiroaki Kataoka

**Affiliations:** 1 Department of Urology, Faculty of Medicine, University of Miyazaki, 5200 Kihara, Kiyotake, Miyazaki, 889-1692 Japan; 2 Oncopathology and Regenerative Biology Section, Faculty of Medicine, University of Miyazaki, Miyazaki, Japan; 3 Laboratory of Molecular Cell Biology Analysis, Faculty of Medicine, University of Miyazaki, Miyazaki, Japan

**Keywords:** Matriptase, MET, Bone metastasis, RCC, Osteoclast

## Abstract

High MET expression in renal cell carcinoma (RCC) and MET activation in bone metastases are reportedly important in progression of several cancers. To find new treatment targets in bone metastasis, we immunohistochemically analyzed expression levels of MET and matriptase (specific cellular activator of hepatocyte growth factor). We obtained nephrectomy specimens from 17 RCC patients with metastasis, and bone metastases specimens from 7 RCC patients who underwent metastasectomies, and who were treated at our hospital between 2008 and 2012. We tested the samples with anti-human MET polyclonal antibody and anti-human matriptase polyclonal antibody, and compared postoperative overall survival (OS) rates between positive and negative groups. High MET expression was seen at primary sites in 8/17 (47 %) nephrectomy specimens, and 6/7 (86 %) bone specimens. Matriptase was expressed in 6/17 (35 %) nephrectomy specimens, and all 7 (100 %) bone specimens. Interestingly, matriptase was strongly expressed in osteoclasts of 5/7 bone specimens. Postoperative OS rate was significantly higher in the MET^−^ group than the MET^+^ group. The high MET and matriptase expression seen in RCC cells in bone metastasis accompanied by matriptase expression in osteoclasts indicates their importance in bone metastasis.

## Introduction

Renal cell carcinoma (RCC) is the most common kidney malignancy [1, 2]. Although most patients without metastasis can be cured by nephrectomy alone, approximately 30 % of RCC patients have metastasis, and nephrectomy is usually not curative for these patients [1, 2]. In RCC patients with metastasis, bone is the second most common metastatic site after lung. Bone metastasis is difficult to control, and is a predictor of poor prognosis [1, 2]. In addition, osteolytic metastasis significantly affects patients through skeletal-related events (SREs), such as pathological fractures, spinal cord compression or hypercalcemia. Unfortunately, the efficacy of recent multimodal treatments, including surgical resection, radiation, osteoclast inhibition, and targeted therapy for vascular endothelial growth factor (VEGF) or mammalian target of rapamycin (mTOR) pathway toward the bone metastasis, is not enough.

Most RCC cases are classified as clear-cell type (conventional RCC); the next most common classification is papillary RCC. Germline-inactivating mutations in the *VHL* tumor suppressor gene and activating mutations in the *MET* gene lead to von Hippel–Lindau disease and hereditary type-1 papillary RCC, respectively [3, 4]. Whereas the *VHL* tumor suppressor gene is inactivated by somatic mutation or promoter methylation in most clear-cell RCC, somatic *MET*-activating mutations are not apparent in sporadic clear-cell RCC [4]. However, increased expression of MET and hepatocyte growth factor (HGF), and enhanced activation of pro-HGF have been seen in clear-cell RCC [5–7]. Moreover, poor prognosis, and overexpression of HGF, cellular activator of pro-HGF (hepsin) and MET are reportedly correlated, which indicates the importance of HGF-dependent *MET* activation in progression of clear-cell RCC [5–7]. Therefore, cell surface activation of pro-HGF might be important in conventional RCC. Several researchers have studied primary-site specimens, but Weber et al. [8] published the only study of high MET expression in an RBMI cell line from a RCC bone metastasis.

MET is a high-affinity receptor tyrosine kinase of HGF, which is a well-known multifunctional growth factor. The HGF/MET signaling axis is apparently involved in tumor progression [9]. HGF is secreted as an inactive single-chain precursor (pro-HGF), which lacks biological activity and thus requires proteolytic activation for conversion to an active two-chain form. Matriptase [a member of the type-2 transmembrane serine protease (TTSP) family] is the most efficient known cellular activator of pro-HGF. Matriptase has been proposed to initiate signaling and proteolytic cascades through its ability to activate pro-urokinase and protease-activated receptor 2 (PAR2) [10], and is reported to efficiently activate macrophage-stimulating protein (MSP) and platelet-derived growth factors (PDGF) C and D [11, 12]. Matriptase expression has been reported in breast, prostate, ovarian and cervical cancer and RCC [10, 11, 13, 14] and its expression is described as correlating with tumor severity in breast, prostate cancer, and RCC [5, 10, 13]. However, matriptase expression in bone metastasis has not been examined.

Here, we immunohistochemically analyzed expression of MET and matriptase protein in RCC primary sites and bone metastases, and evaluated their clinical relevance.

## Patients and methods

This is a retrospective study in which the clinical data were obtained from clinical records and the tumor specimens were from paraffin-embedded blocks. The experimental protocol was approved by the Ethical Review Committee of Miyazaki University. Kidney specimens were obtained from 17 RCC patients with metastasis who received radical nephrectomies at our institution from 2008 to 2012; they included 14 clear-cell type RCC, 2 type-2 papillary RCC and 1 chromophobe RCC. We also obtained 7 bone metastasis specimens from patients with advanced RCC who underwent metastasectomies at our hospital between 2008 and 2012. Patients’ mean age was 62 ± 11 years [standard deviation (SD); range: 44–79 years); the male/female ratio was 17/3.

### Immunohistochemistry and analysis

Formalin-fixed paraffin-embedded sections were prepared according to routine method. The specimens of bone metastasis were subjected to a decalcification procedure using 10 % ethylenediamine-tetra-acetic acid (pH 7.2) for 12–24 h, and were used for hematoxylin and eosin stain, and immunohistochemistry. For immunohistochemistry, sections were processed for antigen retrieval (microwave in 10 mM citrate buffer, pH 6.0 for 10 min), followed by treatment with 3 % H_2_O_2_ in methanol for 10 min and washed in tris-buffered saline (TBS) twice. After blocking in 3 % bovine serum albumin and 5 % goat serum in phosphate-buffered saline for 2 h at room temperature, the sections were incubated with primary antibodies overnight at 4 °C. Anti-human MET rabbit polyclonal antibody was purchased from Immuno-Biological Laboratories (Gunma, Japan) and anti-human matriptase polyclonal antibody was from LifeSpan Biosciences (Seattle, WA, USA). Negative controls did not include the primary antibody. Sections were then washed in TBS and incubated with Envison-labelled polymer reagent (DAKO) for 30 min at room temperature. Sections were exposed with nickel, cobalt-3, 3-diaminobenzidine (Immunopure Metal Enhanced DAB Substrate Kit; Piece, Rockford, IL, USA), and counterstained with hematoxylin.

Immunoreaction staining intensity was judged by percentage of RCC cells in which the cancer cell membranes were stained (e.g., if 80 out of 100 cells were stained, staining was 80 %): staining of >80 %, strongly positive (2+); 20–80 %, positive (1+); 5–20 %, weakly positive (±); < 5 %, negative (−). Evaluation was performed by two experienced pathologists. We regarded 2+ and + findings as positive, ± and – findings as negative.

### Statistical analysis

Statistical parameters were assessed using SPSS statics, version 17.0 (SPSS, Chicago, IL, USA). For analysis of follow-up data, overall survival (OS) was calculated by Kaplan–Meier method; survival distributions were compared by log-rank test.

## Results

**Fig. 1:**
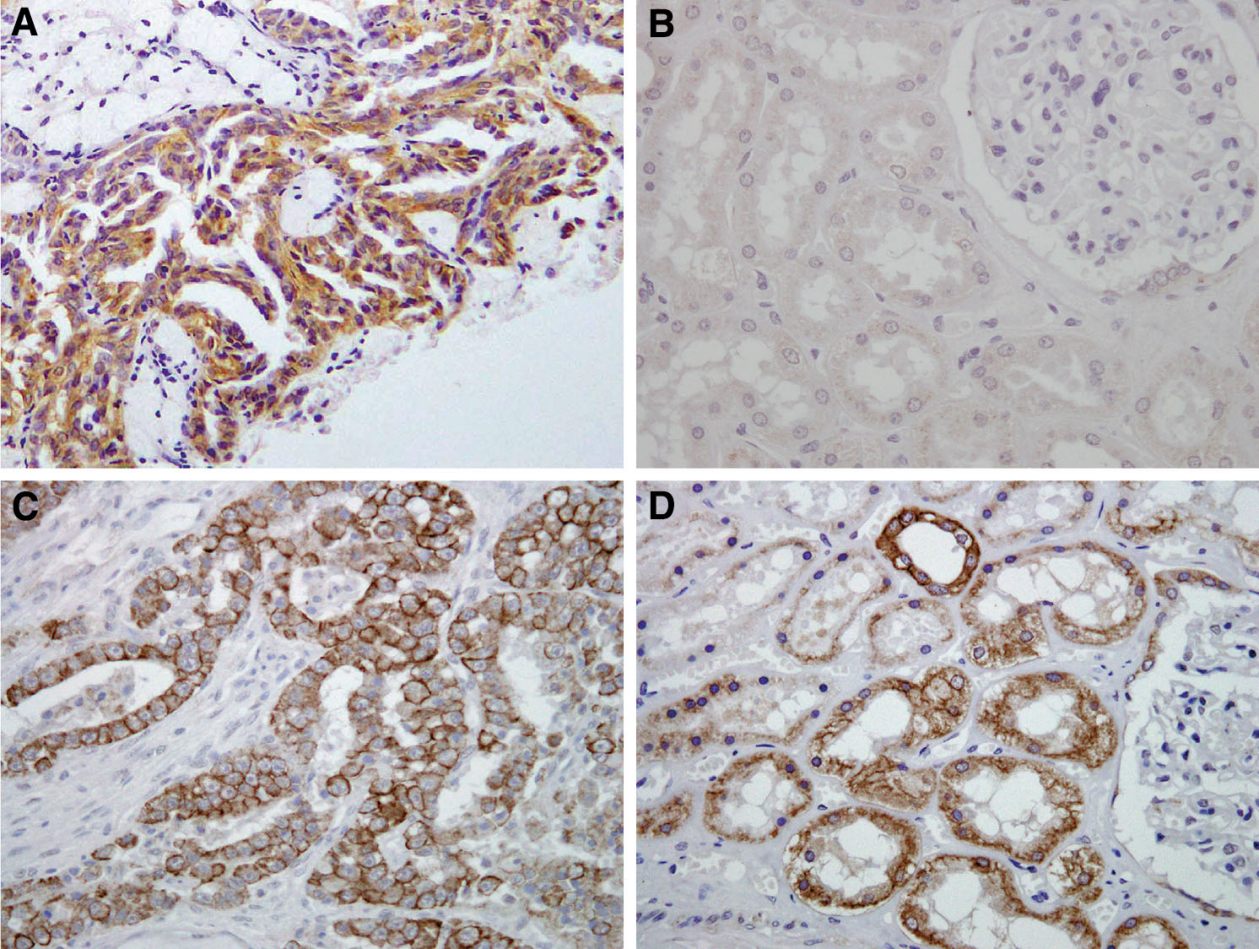
Immunohistochemical staining of MET (**a**, **b**) and matriptase (**c**, **d**). Although the type-1 papillary RCC cells were stained with anti-MET antibody (**a**), normal renal tubules and glomeruli were MET^−^ (**b**). In normal kidney, renal tubules were partially matriptase^+^; however, the glomeruli were not stained with anti-matriptase antibody (**d**). Surface of the cancer cells were strongly immunostained for matriptase, whereas stromal cells were negative (**c**)

**Fig. 2:**
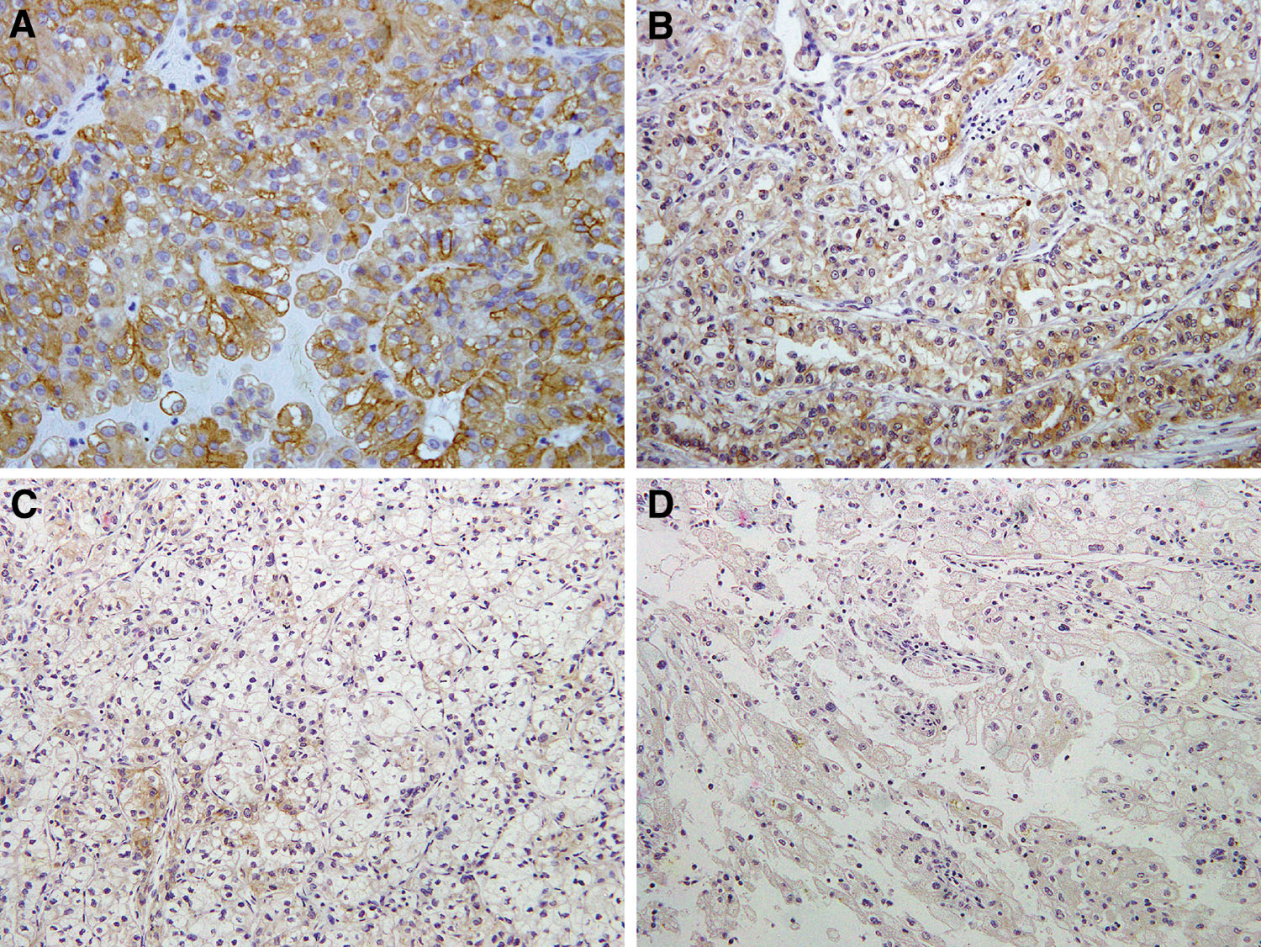
Representative result of MET immunoreactivity in RCC. Tumor cells from 8 (47 %) primary specimens and 6 (86 %) bone metastases were stained strongly positive (2+) (**a**) to positive (1+) (**b**), whereas in nine primary specimens (53 %) and 1 bone metastasis (14 %), tumor cells were stained slightly positive (±) (**c**) or negative (−) (**d**)

**Fig. 3:**
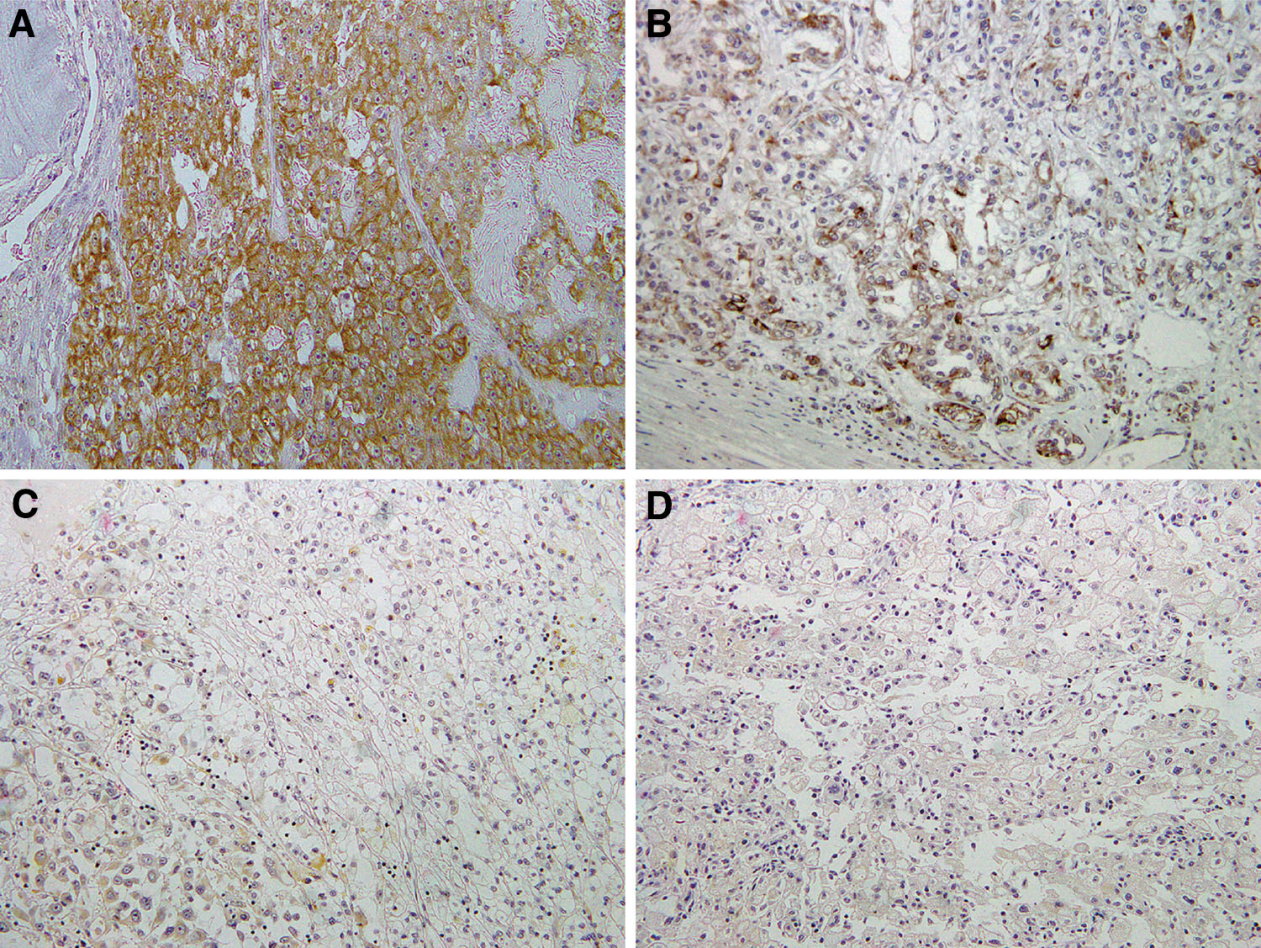
Representative result of matriptase immunoreactivity in RCC. Cancer cells from primary sites in six primary specimens (35 %) and seven bone metastases (100 %) were strongly positive (2+) (**a**) or positive (+) (**b**), and in nine primary specimens (65 %), cancer cells were slightly positive (±) (**c**) or negative (−) (**d**)

Immunohistochemical appearance is shown in Fig. 1. In the normal kidneys, MET was not expressed in renal tubules, collecting ducts or glomeruli (negative control) (B); however, strong expression was observed in type-1 papillary RCC, which was assigned as a positive control (A). However, matriptase was highly expressed in normal renal tubules (positive control), while the glomeruli were not stained (negative control) (D). These findings were consistent with the findings of Jin et al. [14]. Predominant staining of matriptase was seen on cancer cell membranes of cancer cells (Fig. 1c), whereas cancer-associated stromal cells were matriptase^−^. These staining patterns were confirmed in primary-site and bone metastasis specimens (Figs. 2, 3).

**Fig. 4:**
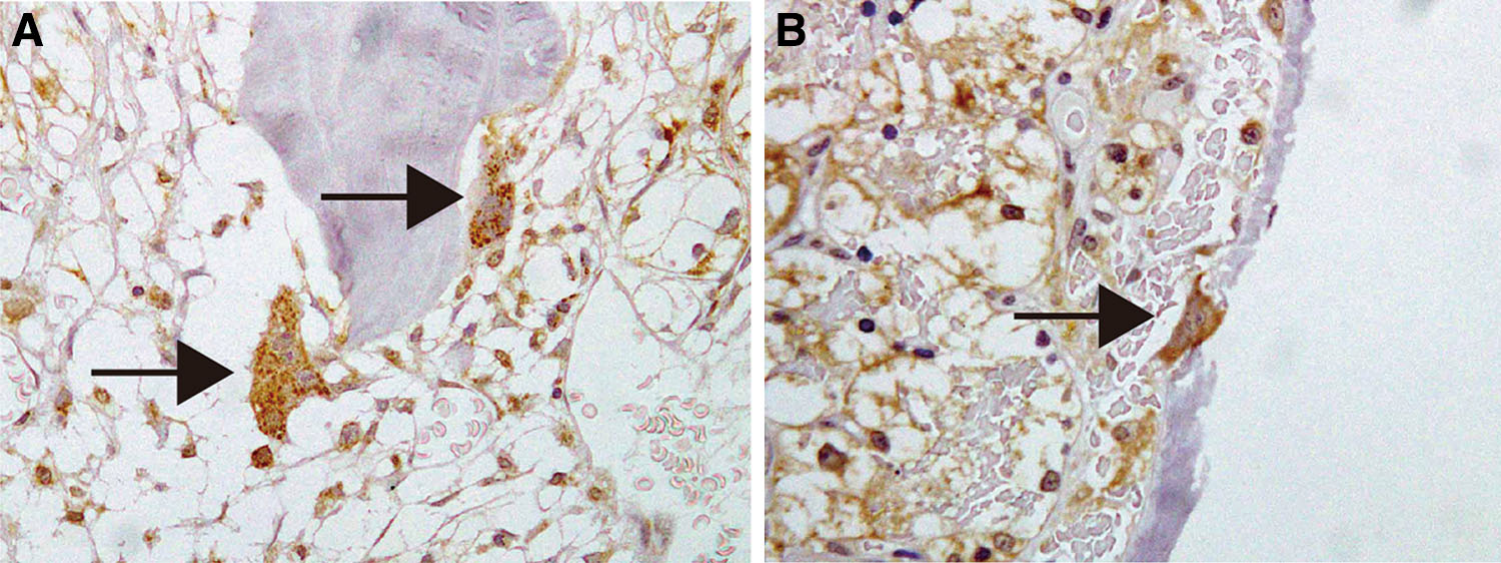
Immunohistochemical staining of matriptase in osteoclasts (**a**, **b**). In five cases (71 %) of bone metastasis, cellular surface and cytoplasm of the osteoclasts were stained positive with anti-matriptase antibody. *Arrowhead*: osteoclasts (**a**, **b**)

Twelve patients had metastasis at diagnosis (Table 1), and metastasis metachronously occurred after nephrectomy in eight patients. In four patients, we could analyze MET and matriptase expression in both primary sites and bone metastasis. High MET expression was observed at the primary site in 8 of 17 (47 %) kidney specimens, which was consistent with previous reports [5, 6]. On the other hand, MET protein was highly expressed in 6/7 bone metastases (86 %). Although matriptase expression was seen at primary site in 6 of 17 (35 %) specimens, matriptase was significantly expressed in all specimens of bone metastasis. Interestingly, strong matriptase expression in osteoclasts was found in 5/7 specimens of bone metastasis (patients 1, 2, 13, 15, 19; Fig. 4). In the primary-site histopathological classifications, expression of MET was seen in 6 (35 %) and matriptase in 4 (28 %) of 14 clear-cell carcinomas. Both MET and matriptase were highly expressed in all specimens with type-2 papillary RCC. No expression of either was observed in the case of chromophobe RCC.

**Table 1:** Patient characteristics

Patient	Age/sex	Pathological findings			Metastasis	Months after nephrectomy
		pT	Histology	Fuhrman		
1	66/M	3b	Clear	2	S	0
2	73/M	3a	Clear	2	S	0
3	49/M	3b	Clear	2	S	0
4	57/M	2	Clear	2	S	0
5	44/M	3b	Clear	2	S	0
6	61/F	3a	Clear	3	S	0
7	59/F	3a	Clear	2	S	0
8	52/M	4	Clear	2	S	0
9	69/M	3a	Clear	3	S	0
10	61/M	2	Clear	2	S	0
11	75/M	3a	Papillary	2	S	0
12	79/M	3a	Chromo	2	S	0
13	67/F	2	Clear	1	M	168
14	65/M	1b	Clear	2	M	61
15	57/M	1b	Clear	3	M	37
16	54/M	3a	Clear	2	M	48
17	77/M	3a	Clear	2	M	45
18	56/M	3a	Clear	3	M	13
19	44/M	1b	Papillary	3	M	22
20	74/M	3a	Papillary	2	M	21

*M* male, *F* female, *pT* pathological T stage, *Clear* clear-cell renal cell carcinoma (RCC), *Papillary* type-2 papillary RCC, *Chromo* chromophobe RCC, *Fuhrman* fuhrman nuclear grade, *S* metastasis synchronously appeared at diagnosis, *M* metastasis metachronously appeared at diagnosis, *Months after nephrectomy* period from nephrectomy until metastasis appeared, in months

**Fig. 5:**
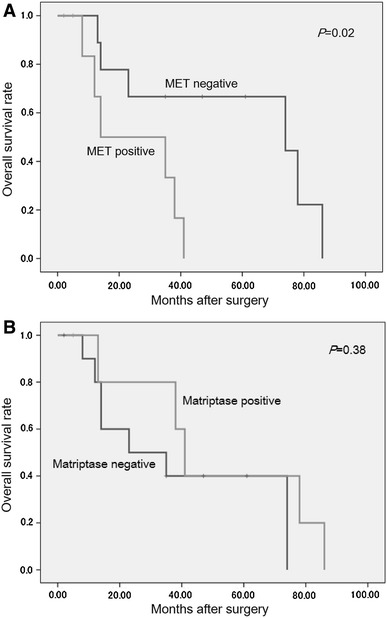
Comparison of overall survival (OS) rates of RCC patients in MET (**a**) or matriptase (**b**) positive and negative (at primary site) groups. A The OS rate of MET^−^ group was significantly better than that of the MET^+^ group. B Matriptase expression and OS were not significantly associated

Next, we examined whether primary-site expression of MET or matriptase was associated with OS after nephrectomy (Fig. 5). Kaplan–Meier analysis showed a significant correlation between MET expression and reduced OS (*P* = 0.02), but no such relationship for matriptase and OS (Table 2).

**Table 2:** Comparative immunoreactivity of MET and matriptase in primary tumor site (kidney) and bone metastases

Patient	Kidney		Bone	
	MET	Matriptase	MET	Matriptase
1	ND	ND	1+	1+
2	±	±	±	2+
3	±	–	1+	2+
4	1+	2+	ND	ND
5	±	–	ND	ND
6	1+	±	ND	ND
7	±	1+	ND	ND
8	1+	–	ND	ND
9	1+	±	ND	ND
10	1+	–	ND	ND
11	2+	1+	ND	ND
12	±	–	ND	ND
13	ND	ND	1+	2+
14	±	1+	2+	1+
15	±	1+	1+	2+
16	±	–	ND	ND
17	±	±	ND	ND
18	2+	±	ND	ND
19	ND	ND	1+	2+
20	1+	1+	ND	ND
Positive	8/17 (47 %)	6/17 (35 %)	6/7 (86 %)	7/7 (100 %)

*Positive* 2 + and 1 + , *Negative* ± and −, *ND* not done

## Discussion

In the present study, we analyzed expressions of both MET and matriptase at primary sites and bone metastases in patients with RCC. As a result, higher MET and matriptase expression were noted at bone metastases than at primary sites. Although this is a retrospective study with few cases, our results indicate the importance of these molecules in bone metastasis. The immunohistochemical appearance of four cases (patients 2, 3, 14, 15), which were comparable at both primary and metastatic sites, especially supports this hypothesis. High MET expression has been reported in bone metastasis: Knudsen et al. [15] reported higher MET expression in bone metastasis than in primary prostate tumors, using immunohistochemical analysis; and Previdi et al. performed in vivo studies and reported that treatment with tivantinib (a MET inhibitor) reduced bone metastasis progression and cancer cell-induced bone destruction with improved survival in breast cancer [16, 17]. Moreover, the significant antitumor efficacy of new tyrosine kinase inhibitor (TKI) XL-184 (cabozantinib maleate), which targets MET and VEGFR-2, for bone metastasis in patients with prostate or breast cancers also shows the importance of HGF/MET signaling in bone metastasis [9, 18].

The deregulation of proteolysis is a well-known hallmark of cancer. Microenvironment protease activity, such as the proteolytic activation of growth factors, degradation of extracellular matrix, and initiation of coagulation cascade, is critically important for cancer cells [9, 20]. The serine proteases that localize to the plasma membrane (including TTSPs) are key factors in cancer invasion [20].

To the best of our knowledge, the present study is the first report to describe matriptase expression in RCC bone metastases, where it is more highly expressed than in the RCC primary sites. As mentioned above, matriptase distinctively processes several substrates. As bone marrow has a great number and amount of growth factors that support cancer survival, PDGFs are thought to be substrates of matriptase. In fact, PDGFRβ, a receptor of PDGF-BB and PDGF-DD, is activated in bone metastasis, and blocking its signaling inhibited growth of breast cancer cells in the bone microenvironment [21].

MSP is another candidate substrate because Receptor d’origine nantais (RON), which is the specific receptor of MSP, has been confirmed to be highly expressed in osteoclasts, and activation of osteoclast in vitro by breast cancer cell-induced MSP has been reported [22]. In addition, breast cancer patients with high MSP/matriptase/RON expression showed significant osteolytic bone metastasis compared with patients without these molecules [23]. In the present study, matriptase expression was often seen in both cancer cells and osteoclasts. Indeed, all bone metastases in this study appeared to be clinically osteolytic. In addition, activation of PAR2 is reportedly significant for normal osteoblast and osteoclast differentiation [24]. PAR2 is a proteolytic target for matriptase, which may have a strong association with matriptase in osteoclasts of bone specimens. Our findings and previous reports support the important role of matriptase in osteolytic bone metastasis; however, further examination is required to clarify the mechanism.

In conclusion, high MET and matriptase expression was found immunohistochemically in RCC cells that had metastasized to bone and was accompanied by matriptase expression in osteoclasts, which implies a role for these molecules in bone metastasis.

## Abbreviations

HGF: Hepatocyte growth factor
MSP: Macrophage-stimulating protein
PDGF: Platelet-derived growth factor
RCC: Renal cell carcinoma
RON: Receptor d’origine nantais
VHL: Von Hippel–Lindau
